# CHA_2_DS_2_-VA and non-CHA_2_DS_2_-VA components as predictors of mortality after cavotricuspid isthmus ablation: a clinical tool for long-term risk stratification

**DOI:** 10.3389/fcvm.2026.1884992

**Published:** 2026-07-20

**Authors:** Kamilla Luca Dávid, Előd János Zsigmond, Manuella Bogdan, Balázs Polgár, Nikolett Vigh, Zalán Gulyás, Judit Papp, Emese Tóth-Zsámboki, Gábor Zoltán Duray

**Affiliations:** 1 Department of Cardiology, Central Hospital of Northern Pest - Military Hospital, Budapest, Hungary; 2 Doctoral School of Clinical Medicine, Cardiovascular Medicine and Research Division, Semmelweis University, Budapest, Hungary; 3 Heart and Vascular Center, Semmelweis University, Budapest, Hungary

**Keywords:** atrial flutter, cavotricuspid isthmus ablation, CHA_2_DS_2_-VA score, mortality, risk stratification, survival prediction

## Abstract

**Introduction:**

Typical atrial flutter (AFL) is associated with considerable morbidity and mortality despite high procedural success rates of cavotricuspid isthmus (CTI) ablation. While the CHA₂DS₂-VA score is widely used for thromboembolic risk assessment in atrial fibrillation, its prognostic value for long-term survival after CTI ablation remains unclear. Our objective therefore was to evaluate the association between the CHA₂DS₂-VA score, its individual components, and non-CHA₂DS₂-VA comorbidities with all-cause mortality following CTI ablation for typical AFL.

**Methods:**

We retrospectively analyzed clinical and mortality data from 249 consecutive patients who underwent successful CTI ablation between July 2017 and December 2021. Kaplan-Meier survival analysis, Cox regression, and time-dependent ROC analyses were performed to evaluate predictors of all-cause mortality.

**Results:**

During a mean follow-up of 1771 ± 635 days, 27% of patients died. Mortality increased progressively with higher CHA₂DS₂-VA scores, corresponding to a stepwise decline in survival (log-rank *p* < 0.001). A CHA₂DS₂-VA score ≥3 was identified as the optimal threshold for mortality prediction, with annual mortality rates of 1.4% versus 9.9% in patients with scores <3 and ≥3, respectively (*p* < 0.001). The CHA₂DS₂-VA score demonstrated good discriminative ability (c-index = 0.73). In multivariable analysis, the CHA₂DS₂-VA score remained an independent predictor of all-cause mortality (HR 1.48, 95% CI 1.26 -1.75, *p* < 0.001). Among non-CHA₂DS₂-VA variables, pulmonary disease and obstructive sleep apnoea independently predicted adverse long-term survival, whereas female sex was not significantly associated with mortality.

**Discussion:**

The CHA₂DS₂-VA score is an independent predictor of long-term all-cause mortality after CTI ablation for typical AFL, extending its clinical utility beyond thromboembolic risk assessment. Incorporation of selected non-CHA₂DS₂-VA comorbidities may further refine risk stratification and support individualized follow-up strategies.

## Introduction

Atrial arrhythmias are among the most common cardiac rhythm disorders encountered in clinical practice. Typical atrial flutter (AFL) is characterized by a macro–reentrant circuit within the right atrium. Although AFL is generally considered less malignant than atrial fibrillation (AF), particularly regarding thromboembolic risk, emerging evidence indicates that the overall risk profile of AFL—including mortality and progression to AF—remains clinically significant. While several studies have reported lower stroke rates in AFL compared with AF in selected cohorts, other outcomes, such as all-cause mortality may not be reduced, especially in populations undergoing AFL ablation (1–3). Cavotricuspid isthmus (CTI) ablation is the first-line therapy for typical AFL with high procedural success and low recurrence rates (4). Despite excellent acute efficacy, long-term prognosis following ablation varies substantially and is influenced by underlying comorbidities and cardiovascular risk profiles (5, 6). Recent evidence suggests that patients with AFL may share similar thromboembolic and cardiovascular risk patterns with those seen in AF, particularly when evaluated using established clinical risk stratification tools such as the CHA_2_DS_2_-VASc score (7–9). Furthermore, many patients who undergo CTI ablation subsequently develop AF or experience other adverse cardiovascular events (5, 10).

Originally developed for estimating thromboembolic risk in AF, the CHA_2_DS_2_-VASc score is recommended by current European Society of Cardiology guidelines as the standard clinical tool for stroke and systemic embolism risk assessment in both AF and AFL patients (11). Beyond its role in guiding anticoagulation, several studies have demonstrated that the CHA_2_DS_2_-VASc score also predicts all-cause and cardiovascular mortality across diverse cardiovascular populations, including AF and heart failure cohorts (12–14).

However, outside the context of AF, the prognostic value of the CHA_2_DS_2_-VASc score—or its recently proposed, guideline-supported modification, the CHA_2_DS_2_-VA score—for predicting mortality has not been systematically assessed. In patients with typical AFL undergoing CTI ablation, its performance remains insufficiently characterized. Prior investigations have focused predominantly on thromboembolic or composite cardiovascular outcomes, with limited evaluation of post-ablation survival as an independent endpoint. Given that the CHA_2_DS_2_-VA score removes female sex as a non-independent factor (15–18), its validation as a prognostic tool beyond AF—particularly in typical AFL following CTI ablation—could provide clinically useful insights into long-term risk stratification. Moreover, the relative contribution of individual CHA_2_DS_2_-VA components and other comorbidities to long-term mortality in this population remains unclear.

To address this knowledge gap, we conducted an observational study of patients with typical AFL who underwent successful CTI ablation, aiming to evaluate the impact of CHA_2_DS_2_-VA score components and additional clinical risk factors on long-term survival.

## Materials and methods

### Patient enrollment

We conducted a retrospective observational cohort study including 249 consecutive patients who underwent CTI ablation for typical AFL at our centre between July 2017 and December 2021. A CHA_2_DS_2_-VA score was calculated at the time of ablation and evaluated as a predictor of all-cause mortality. In addition, we assessed whether each individual component of the score independently predicted survival. We also examined the potential independent associations of comorbidities not included in the CHA_2_DS_2_-VA score, including cardiac implantable electronic devices (CIEDs), obstructive sleep apnea syndrome (OSAS), valvular heart disease, thyroid dysfunction, gastroesophageal reflux disease, dyslipidemia, pulmonary disorders (including chronic obstructive pulmonary disease, asthma bronchiale, pulmonary fibrosis, emphysema, or chronic respiratory failure and related chronic pulmonary conditions) and renal impairment. As female sex is no longer included in the CHA_2_DS_2_-VASc score and current guidelines recommend the use of the CHA_2_DS_2_-VA score, female sex was additionally evaluated as a potential independent predictor of mortality. Baseline demographic and clinical characteristics, as well as procedural information, were extracted from electronic medical records and procedural databases.

All patients provided written informed consent prior to catheter ablation. The study was approved by the Institutional Ethics Committee and conducted in accordance with the Declaration of Helsinki. This study was reported in accordance with the Strengthening the Reporting of Observational Studies in Epidemiology (STROBE) guidelines.

### Follow-up

Information on mortality and the last available clinical follow-up was obtained from the National eHealth Infrastructure (EESZT) system. Approximately one-third of patients were followed in the outpatient clinic of the operating centre, while the remainder received follow-up care in external institutions. The final follow-up date was February 18, 2025. Vital status was available for all patients at the end of follow-up, and no patients were lost to follow-up for mortality assessment.

### Statistical analysis

All statistical analyses were performed using SPSS software (IBM SPSS Statistics versions 19.0 and 22.0), TIBCO Statistica, and R version 4.3.2 (R Core Team, 2024) using RStudio. All statistical tests were two-tailed, and a *p*-value < 0.05 was considered statistically significant. The Shapiro–Wilk test was used to assess normality because of its high sensitivity for detecting departures from normality and its broad applicability across a wide range of sample sizes. Normally distributed continuous variables are presented as mean ± standard deviation (SD), whereas skewed variables are expressed as median (interquartile range). Categorical variables are presented as counts and percentages. Between-group comparisons were performed using the independent-samples Student's *t*-test for normally distributed continuous variables and the Mann–Whitney *U*-test for non-normally distributed continuous variables. Categorical variables were compared using the Chi-square test or Fisher's exact test, as appropriate. The association between individual CHA_2_DS_2_-VA scores and all-cause mortality was evaluated using Spearman's rank correlation analysis, with the corresponding *p*-value reported. Additionally, aggregated mortality rates by CHA_2_DS_2_-VA score categories were analyzed using ordinary least squares (OLS) linear regression. Survival analyses were conducted using the Kaplan–Meier method, with comparisons made using the log-rank test. *post-hoc* pairwise log-rank comparisons between CHA_2_DS_2_-VA score categories were performed using Holm–Bonferroni adjustment for multiple testing. Univariate Cox proportional hazards models were used to evaluate associations between clinical variables and all-cause mortality, generating hazard ratios (HRs) with 95% confidence intervals (CIs). Variables with *p* < 0.10 in univariate analysis were entered into a multivariable Cox regression model to identify independent predictors, excluding those already incorporated into the CHA_2_DS_2_-VA score to avoid collinearity.

Time-dependent receiver operating characteristic (ROC) analyses were performed to evaluate the discriminatory ability of the CHA_2_DS_2_-VA score, with optimal cut-off values identified using the highest Youden index. Discrimination was further quantified using the concordance index (C-statistic).

## Results

### Baseline characteristics

A total of 249 patients were included in the final analysis, comprising 195 (78%) females and 54 (22%) males (Table 1). The mean age was 66 ± 12.6 years, and the mean BMI was 29.7 ± 5.6 kg/m^2^. Left ventricular ejection fraction (LVEF) averaged 51.3 ± 13.8%. All patients underwent CTI ablation: 111 (45%) with zero-fluoroscopy (ZF), 122 (49%) with conventional fluoroscopy, and 16 (6%) with an initial ZF approach completed using fluoroscopy.

**Table 1:** Baseline demographic and clinical characteristics of the study population.

Patient Characteristics	Total population *n* = 249	CHA_2_DS_2_VA score (0–1-2) Group 1 *n* = 118	CHA_2_DS_2_VA score (≥3) Group 2 *n* = 131	*p*-values
Gender (female/male), n	195/54 (78/22%)	90/28 (76/24%)	105/26 (80/20%)	0.458
Death at control time, *n* (%)	67 (27%)	8 (7%)	59 (45%)	**0**.**000**
Mortality per year	5.6%	1.4%	9.9%	**0**.**000**
Mean of age (years)	66 ± 12.6	59 ± 10.4	72 ± 10.7	**0**.**000**
BMI (kg/m^2^)	29.7 ± 5.6	29 ± 5.4	30 ± 5.7	0.138
EF%	51.3 ± 13.8	57 ± 12	46 ± 14	**0**.**000**
Follow-up (days)	1,771 ± 635	1,890 ± 530	1,664 ± 702	**0**.**043**
CHA_2_DS_2_VA score variables, *n* (%)				
Congestive heart failure	59 (24%)	8 (7%)	51 (39%)	**0**.**000**
Hypertension	206 (83%)	79 (67%)	127 (97%)	**0**.**000**
Age =>75	60 (24%)	2 (2%)	58 (44%)	**0**.**000**
Diabetes mellitus	71 (29%)	9 (8%)	62 (47%)	**0**.**000**
Stroke/TIA/thromboembolic event	29 (12%)	1 (1%)	28 (21%)	**0**.**000**
Vascular disease				
(AMI, PAD, aortic plaque)	51 (21%)	3 (2.5%)	48 (37%)	**0**.**000**
Age (65–74 years)	82 (33%)	34 (29%)	48 (37%)	0.119
Non—CHA_2_DS_2_VA score variables, *n* (%)				
CIED	28 (11%)	9 (8%)	19 (15%)	0.064
Valvular disease	16 (6%)	4 (3%)	12 (9%)	0.064
Ischaemic heart disease	77 (31%)	18 (15%)	59 (45%)	**0**.**000**
Pulmonary disorder	32 (13%)	10 (8.5%)	22 (17%)	**0**.**037**
OSAS	10 (4%)	3 (2.5%)	7 (5%)	0.213
Thyroid gland disease	32 (13%)	12 (10%)	20 (15%)	0.156
Hypothyreosis	20 (8%)	7 (6%)	13 (10%)	0.178
Hyperthyreosis	12 (5%)	5 (4%)	7 (5%)	0.446
GERD	21 (8%)	7 (6%)	14 (11%)	0.131
Dyslipidaemia	34 (14%)	15 (13%)	19 (15%)	0.412
Renal failure	15 (6%)	3 (2.5%)	12 (9%)	**0**.**025**
Stroke	12 (5%)	1 (1%)	11 (8%)	**0**.**005**
Preoperative AF	96 (39%)	45 (38%)	51 (39%)	0.501
New onset AF	8 (3%)	3 (2.5%)	5 (4%)	0.420
Preoperative ECG				
Sinus rhythm	106 (43%)	62 (53%)	44 (34%)	**0**.**002**
AF	8 (3%)	5 (4%)	3 (2%)	0.305
AFL	131 (53%)	49 (41%)	82 (63%)	**0**.**001**
PM	3 (1%)	1 (1%)	2 (1.5%)	0.540
other	1 (0.4%)	1 (1%)	0	0.474
RBBB	41 (16.5%)	19 (16%)	22 (17%)	0.510
LBBB	23 (9%)	7 (6%)	16 (12%)	0.067
LAH	10 (4%)	4 (3%)	6 (4.5%)	0.441

The patient cohort was stratified into two groups based on median distribution of CHA_2_DS_2_-VA scores: Group 1 included patients with a score of 0–2 (below median), while Group 2 included those with a score ≥3 (equal or above median). Due to design of CHA_2_DS_2_-VA score system, age differed significantly between patient groups. Mortality rate was 6.4-fold higher in patients with CHA_2_DS_2_-VA score above/equal median. Among non-CHA_2_DS_2_-VA clinical variables, renal failure, pulmonary disease, and ischemic heart disease were also significantly more prevalent in the higher-score group.

AF, atrial fibrillation; AFL, atrial flutter; AMI, acute myocardial infarction; BMI, body mass index; CHA_2_DS_2_-VA, congestive heart failure, hypertension, age (≥75), diabetes mellitus, stroke/thromboembolic event, vascular disease, age (65–74); CIED, cardiac implantable electronic device; ECG, electrocardiogram; EF, ejection fraction; GERD, gastroesophageal reflux disease; LAH, left anterior hemiblock; LBBB, left bundle branch block; OSAS, obstructive sleep apnea syndrome; PAD, peripheral artery disease; PM, pacemaker; RBBB, right bundle branch block.

Bold values indicate statistically significant differences (*p* < 0.05).

The cohort displayed a high burden of CHA_2_DS_2_-VA risk components: 24% had heart failure, 83% hypertension, 29% diabetes mellitus, 5% prior stroke, 12% previous thromboembolic event, and 21% vascular disease.

### Mortality

#### Association between CHA_2_DS_2_-VA score and mortality

**Figure 1:**
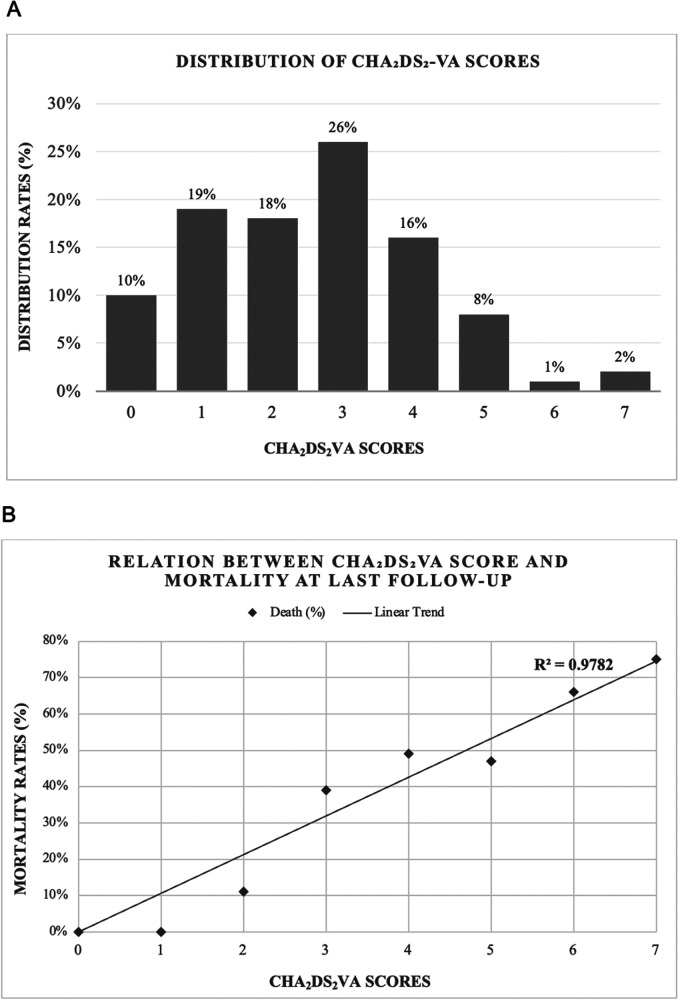
CHA_2_DS_2_-VA score distribution and mortality relationship. **(A)** Distribution of CHA_2_DS_2_-VA scores in the total population. **(B)** Relation between CHA_2_DS_2_-VA score and mortality. Each point represents the observed mortality for the corresponding score. The solid line indicates the linear trend (R^2^ = 0.978), showing a strong positive association between higher CHA_2_DS_2_-VA scores and mortality.

Figure 1A illustrates the distribution of CHA_2_DS_2_-VA scores in the study population, while Figure 1B demonstrates how increasing CHA_2_DS_2_-VA score was strongly associated with higher category-level mortality (R^2^ = 0.978). At the individual patient level, CHA_2_DS_2_-VA score showed statistically significant positive correlation with mortality (*ρ* = 0.423, *p* < 0.001).

The median CHA_2_DS_2_-VA score in the cohort was 3. For descriptive baseline comparison, the study population was divided into two groups according to values below or above/equal to the median (Group 1 and Group 2, respectively). As expected, most CHA_2_DS_2_-VA score components were significantly more frequent in Group 2, except for intermediate age (65–75 years) (Table 1).

To determine the optimal cut-off point for mortality prediction, ROC curve analysis using the Youden index was performed. This analysis identified a CHA_2_DS_2_-VA score of 3 as the optimal threshold, yielding the highest Youden index (0.485). Notably, this cut-off also provided the strongest separation of Kaplan–Meier survival curves, with a highly significant log-rank test (*p* < 0.001).

#### Survival analysis and time-dependent discriminatory performance

Mean follow-up duration was 1,771 ± 635 days. Overall mortality was 27%, corresponding to 5.6% yearly mortality. Causes of death were distributed as follows: 15% cardiac death (sudden 7.5%, non-sudden 7.5%), 53.5% non-cardiac death (infection-related 30%, malignancy-related 19%, other non-cardiac 4.5%), 4.5% stroke, and 27% unknown.

End-of-follow-up mortality was 7% in Group 1 vs. 45% in Group 2. Annual mortality was 7.1-fold higher in patients with a CHA_2_DS_2_-VA score ≥3 (1.4% vs. 9.9%) (Table 1).

**Figure 2:**
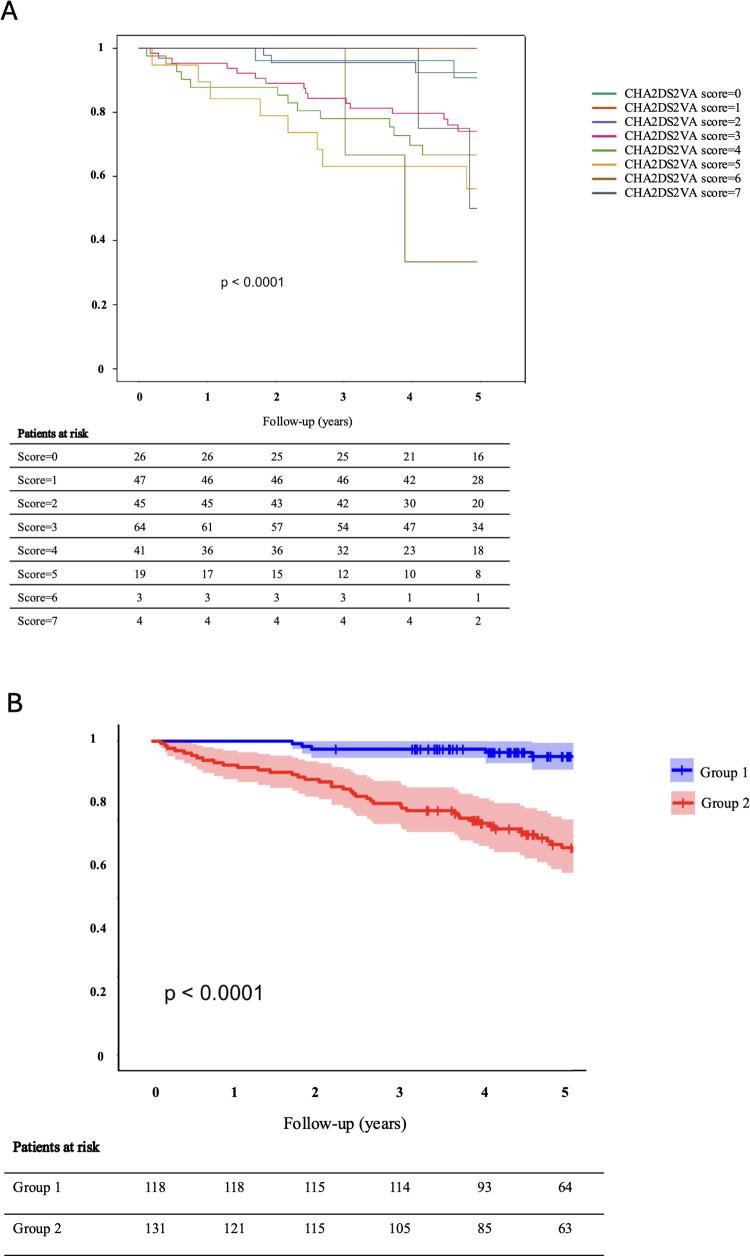
Survival analyses according to CHA_2_DS_2_-VA score. **(A)** Kaplan–Meier survival curves stratified by CHA_2_DS_2_-VA score. Kaplan–Meier estimates of all-cause mortality according to CHA_2_DS_2_-VA score categories. Higher CHA_2_DS_2_-VA scores were associated with progressively reduced survival during follow-up (log-rank *p* < 0.0001). The number of patients at risk for each score category at yearly intervals is shown below the graph. **(B)** Kaplan–Meier survival curves according to CHA_2_DS_2_-VA risk groups. Kaplan–Meier estimates of all-cause mortality in patients stratified into two groups based on the median CHA_2_DS_2_-VA score (Group 1: CHA_2_DS_2_-VA score <3; Group 2: CHA_2_DS_2_-VA score ≥3). Patients with higher CHA_2_DS_2_-VA scores exhibited substantially reduced survival throughout follow-up (log-rank *p* < 0.0001). The number of patients at risk at yearly intervals is shown beneath the figure.

Kaplan–Meier survival curves suggested a graded trend of decreasing survival with increasing CHA_2_DS_2_-VA scores. Patients with higher scores generally showed poorer survival during follow-up compared with those with lower scores (global log-rank test, *p* < 0.001) (Figure 2A). These differences reflect an overall trend across strata. *post-hoc* pairwise log-rank comparisons using Holm-Bonferroni correction identified significant differences primarily between lower and higher CHA_2_DS_2_-VA score categories. The results are provided in Supplementary Table S1. When dichotomized at the Youden-derived threshold, patients with CHA_2_DS_2_-VA scores ≥3 demonstrated significantly higher mortality compared with those with scores <3 (log-rank *p* < 0.001) (Figure 2B).

**Figure 3:**
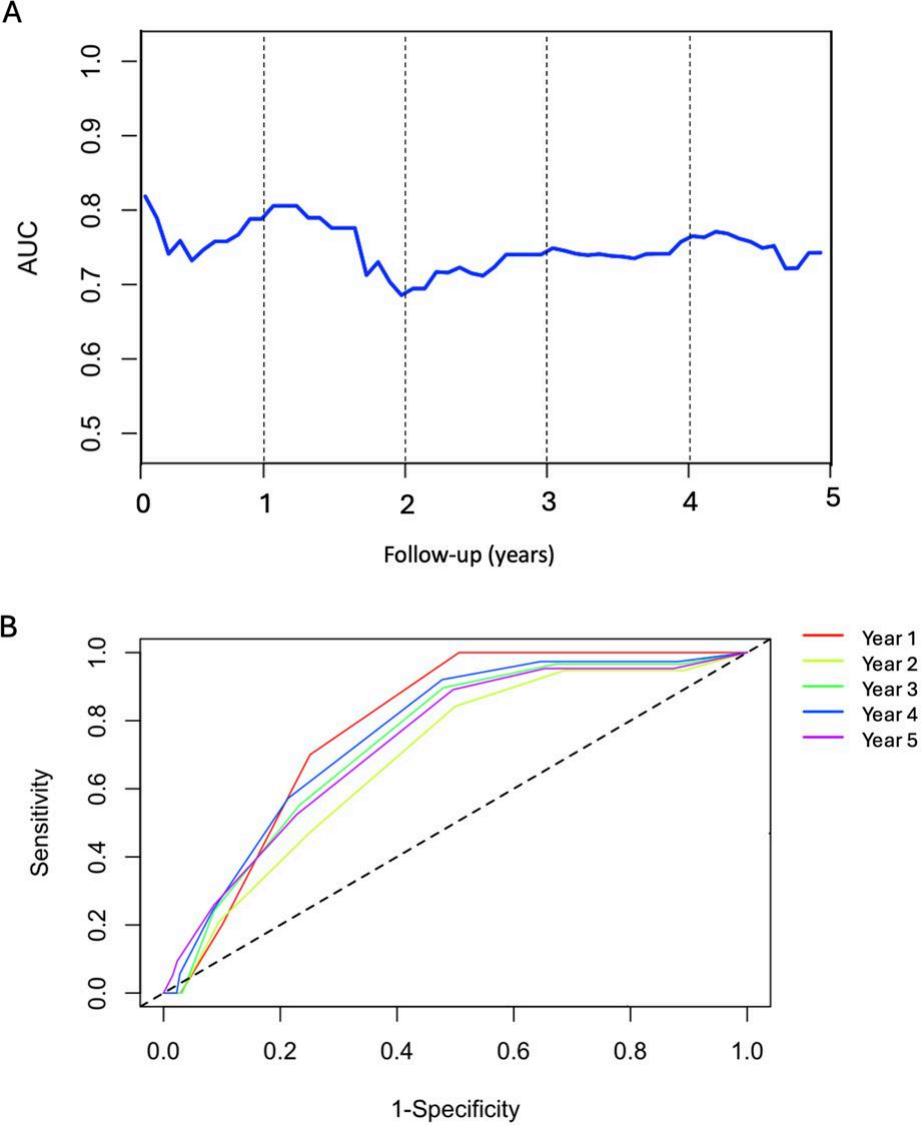
Discriminatory performance of the CHA_2_DS_2_-VA score. **(A)** Time-dependent AUC of the CHA_2_DS_2_-VA score over 5 years of follow-up. Time-dependent area under the ROC curve (AUC) for the CHA_2_DS_2_-VA score across the follow-up period. The score maintained moderate-to-good discriminative ability for predicting all-cause mortality throughout 5 years, with AUC values generally ranging between 0.70 and 0.80. **(B)** Annual ROC curves for mortality prediction during Years 1–5. ROC curves illustrating the discriminative performance of the CHA_2_DS_2_-VA score for predicting 1-year, 2-year, 3-year, 4-year, and 5-year mortality. The score demonstrated consistent predictive ability over time, with similar curve shapes and no major deterioration across the follow-up years.

Time-dependent AUC analysis demonstrated good predictive performance of the CHA_2_DS_2_-VA score after CTI ablation (Figure 3A). Discriminative ability was highest during the first year and remained stable during longer-term follow-up, with AUC values generally ranging between approximately 0.7 and 0.8. Year-specific ROC curves further supported the consistent prognostic performance of the score over time (Figure 3B).

To complement the time-dependent ROC analysis, we calculated the overall concordance index (c-statistic) as a global measure of discrimination across the entire follow-up period.

The CHA_2_DS_2_-VA score achieved a c-index of 0.730, indicating moderate-to-good discriminative performance for mortality prediction.

#### Predictors of mortality—univariate cox regression analysis

##### CHA_2_DS_2_-VA components associated with mortality

**Figure 4:**
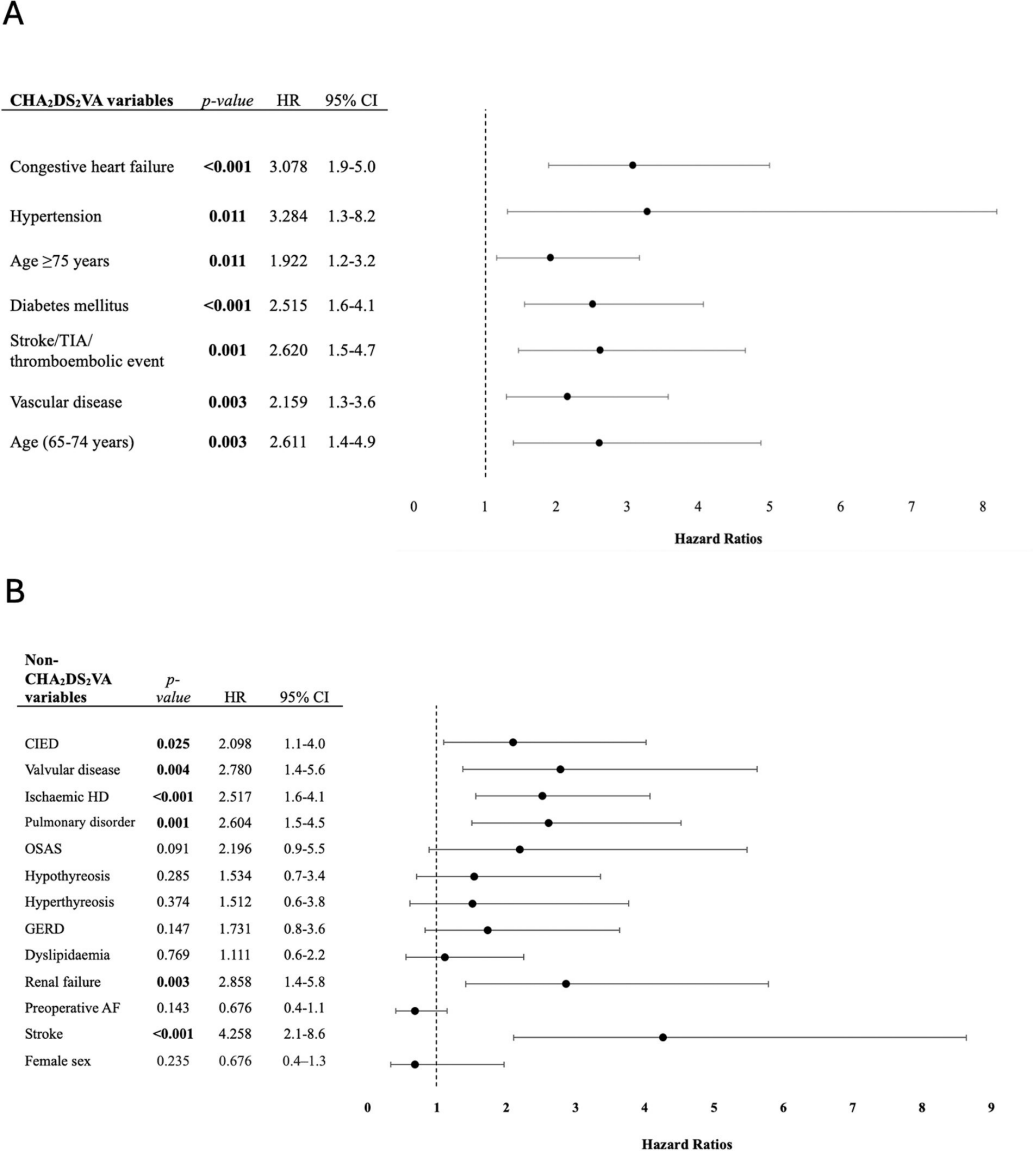
Univariate Cox proportional hazards analyses for predictors of all-cause mortality. **(A)** Hazard ratios for individual CHA_2_DS_2_-VA score components. Univariate Cox regression analysis of each CHA_2_DS_2_-VA component in relation to all-cause mortality. All variables—including congestive heart failure, hypertension, age ≥75 years, diabetes mellitus, prior stroke/TIA/thromboembolic event, vascular disease, and age 65–74 years—were significantly associated with increased mortality risk. Hazard ratios (HRs) with 95% confidence intervals are shown. **(B)** Hazard ratios for non-CHA_2_DS_2_-VA clinical variables. Univariate Cox regression analysis of additional comorbidities not included in the CHA_2_DS_2_-VA score. Several variables—valvular heart disease, ischemic heart disease, pulmonary disorder, renal failure, and presence of a cardiac implantable electronic device (CIED)—were significantly associated with higher mortality. Conditions including OSAS, thyroid dysfunction, GERD, dyslipidaemia, female sex and preoperative atrial fibrillation did not show significant associations. Hazard ratios (HRs) with 95% confidence intervals are displayed.

Cox-regression analysis confirmed significant associations between all individual CHA_2_DS_2_-VA parameters and survival. The presence of congestive heart failure, hypertension, age ≥75 years, diabetes mellitus, prior thromboembolic event, vascular disease and age between 65 and 74 years were all linked to substantially increased mortality during follow-up (Figure 4A).

Within the CHA_2_DS_2_-VA scoring system, when the composite category “stroke/TIA/thromboembolic event” was analysed in more detail, previous stroke alone exhibited the highest hazard ratio (HR = 4.258, *p* < 0.001, 95% CI: 2.10–8.64), indicating the strongest association with mortality. Similarly, the presence of ischemic heart disease was also a powerful predictor, conferring a 2.5-fold increase in mortality risk (Figure 4B).

##### Non-CHA_2_DS_2_-VA predictors of mortality

To evaluate the effect of additional, non-CHA_2_DS_2_-VA clinical variables on survival, Cox univariate analyses were performed for frequent comorbidities documented in the patients' medical histories. The corresponding forest plot is shown in Figure 4B.

Significantly higher mortality was observed in patients with a cardiac implantable electronic device (HR = 2.10, *p* = 0.025, 95% CI: 1.10–4.02), valvular heart disease (HR = 2.78, *p* = 0.004, 95% CI: 1.37–5.62), pulmonary disorder (HR = 2.60, *p* = 0.001, 95% CI: 1.50–4.52), and renal failure (HR = 2.86, *p* = 0.003, 95% CI: 1.41–5.78). Each of these conditions at least doubled the risk of death during follow-up. Female sex was not significantly associated with mortality (HR 0.68, 95% CI 0.35–1.29, *p* = 0.235), supporting the appropriateness of using the CHA_2_DS_2_-VA score rather than the CHA_2_DS_2_-VASc score in this population.

Although OSAS did not reach statistical significance in the univariate analysis (*p* = 0.091), it was included in the multivariate model because of its clinical relevance and borderline significance.

#### Multivariate cox regression analysis

Based on the results of the univariate Cox analyses (*p* < 0.10), the presence of CIED, valvular disease, ischemic heart disease, pulmonary disease, renal failure, OSAS, and the CHA_2_DS_2_-VA score were included as potential predictors in the multivariate Cox proportional hazards model to identify independent determinants of mortality.

The CHA_2_DS_2_-VA score remained a strong independent predictor of all-cause mortality (HR = 1.48, *p* < 0.001, 95% CI: 1.26–1.75). Each point increase in the CHA_2_DS_2_-VA score represents a 1.5-fold rise in mortality risk. In addition, the presence of pulmonary disease (HR = 1.92, *p* = 0.028, 95% CI: 1.07–3.43) and OSAS (HR = 2.66, *p* = 0.044, 95% CI: 1.03–6.87) also emerged as significant independent predictors (Table 2).

**Table 2:** Multivariate Cox proportional hazards analysis for all-cause mortality.

Variables	*p*-value	HR	Lower 95% CI	Upper 95% CI
CIED	0.166	1.632	0.817	3.261
Valvular disease	0.227	1.593	0.748	3.389
Ischaemic heart disease	0.224	1.389	0.817	2.361
Pulmonary disease	**0** **.** **028**	1.919	1.073	3.429
Renal failure	0.636	1.205	0.556	2.613
OSAS	**0**.**044**	2.658	1.028	6.873
CHA_2_DS_2_-VA score	**<0**.**001**	1.484	1.260	1.749

Multivariate Cox regression results evaluating independent predictors of all-cause mortality after CTI ablation. Hazard ratios (HR) with 95% confidence intervals (CI) and corresponding *p*-values are shown for each covariate. Variables included in the model were selected based on univariate analysis (*p* < 0.10). The CHA_2_DS_2_-VA score remained a strong independent predictor of mortality, while pulmonary disease and obstructive sleep apnoea syndrome (OSAS) also demonstrated significant associations with increased risk.

Bold values indicate statistically significant differences (*p* < 0.05).

To assess whether the prognostic value of the CHA_2_DS_2_-VA score was driven predominantly by age, we performed a sensitivity analysis. The age-related components of the score (1 point for age 65–74 years and 2 points for age ≥75 years) were subtracted from the total CHA_2_DS_2_-VA score to construct an age-free score. In a multivariable Cox model including both chronological age and this age-free CHA_2_DS_2_-VA score, the age-free score remained a strong independent predictor of mortality (HR 1.52, *p* < 0.001, 95% CI 1.30–1.79), while age also retained significance (HR 1.04, *p* = 0.001, 95% CI 1.01–1.06). These findings demonstrate that the predictive value of the CHA_2_DS_2_-VA score is not solely attributable to its age component.

## Discussion

In this cohort of patients undergoing CTI ablation for typical atrial flutter, the CHA_2_DS_2_-VA score demonstrated significant prognostic value for long-term all-cause mortality. The strong linear association between increasing score and observed mortality underscores the broader utility of the score, beyond its original purpose of thromboembolic risk assessment in atrial fibrillation.

Using ROC curve analysis and the Youden index, an optimal cut-off value of CHA_2_DS_2_-VA ≥3 was identified for mortality prediction. Patients above this threshold exhibited a 7.1-fold higher yearly mortality rate compared with those with lower scores. These findings suggest that a CHA_2_DS_2_-VA score ≥3 may serve as a simple and clinically meaningful tool for both short- and long-term survival risk stratification in this population. Time-dependent ROC analysis further demonstrated stable predictive performance over the entire follow-up period, with a c-index of 0.730, indicating good discriminative ability. Consistent with this, multivariable Cox regression analysis showed that each one-point increase in the CHA_2_DS_2_-VA score was associated with an approximately 1.5-fold increase in mortality risk. This level of prognostic performance is comparable to that reported in atrial fibrillation cohorts, suggesting that shared pathophysiological mechanisms—such as age-related comorbidity burden, vascular disease, and structural cardiac remodelling—may underlie adverse outcomes in patients with typical atrial flutter as well (19–21). Given the substantial contribution of age to the CHA_2_DS_2_-VA score, we performed a sensitivity analysis to assess whether the observed prognostic value was predominantly age-driven. After subtracting age-related points and incorporating chronological age and the resulting age-free score simultaneously into a multivariable Cox model, the age-free CHA_2_DS_2_-VA score remained a strong independent predictor of mortality. These findings indicate that the discriminative capacity of the CHA_2_DS_2_-VA score cannot be explained by age alone and reflects the prognostic relevance of its non–age-related components.

Data regarding the prognostic role of the CHA_2_DS_2_-VA score in patients with typical atrial flutter remain scarce. The sole available study assessed the predictive ability of the CHADS_2_ and CHA_2_DS_2_-VASc scores for long-term mortality in AFL patients following ablation procedures (22). Recent arrhythmia guidelines recommend the CHA_2_DS_2_-VA score primarily for thromboembolic risk assessment; however, emerging evidence increasingly supports its broader prognostic utility, extending beyond the prediction of cardioembolic ischemic stroke risk (23). Given the established predictive value of the CHA_2_DS_2_-VASc score for various conditions, including heart failure, coronary artery disease, stroke and chronic kidney disease, we hypothesize that the CHA_2_DS_2_-VA score may also serve as an effective clinical tool for predicting outcomes in a broader context (24–29).

Thus, incorporating CHA_2_DS_2_-VA scoring into follow-up protocols for AFL ablation patients might help identify individuals at elevated long-term risk. Patients with CHA_2_DS_2_-VA scores ≥3 might benefit from regular monitoring, including closer surveillance of renal function, pulmonary status, sleep disorders, and cardiovascular health. Particularly, patients with prior stroke or high comorbidity burden, prolonged post-ablation anticoagulation and sustained rhythm surveillance (e.g., periodic Holter, implantable monitors) might be justified, even in the absence of documented AF. However, decisions must be balanced against bleeding risk and patient comorbidity profiles.

Importantly, among non-CHA_2_DS_2_-VA variables, pulmonary disorders and obstructive sleep apnoea syndrome emerged as independent predictors of mortality in the multivariable model, underscoring the prognostic relevance of extracardiac comorbidities in this post-ablation population. Several mechanisms may explain the observed association between pulmonary disease, OSAS, and adverse long-term survival following CTI ablation. Chronic respiratory disorders are frequently accompanied by systemic inflammation, impaired gas exchange, hypoxaemia, and an increased cardiovascular burden, all of which may contribute to adverse long-term outcomes (30). Similarly, OSAS has been associated with repetitive nocturnal hypoxaemia, sympathetic nervous system activation, oxidative stress, and atrial remodelling, all of which may adversely affect long-term cardiovascular results (31). These mechanisms may contribute to recurrent atrial arrhythmias and adverse cardiovascular outcomes despite successful CTI ablation. However, the association between OSAS and mortality should be interpreted cautiously given the small number of affected patients (*n* = 10), borderline significance in univariate analysis, and the relatively wide confidence interval of the multivariable hazard ratio estimate (HR 2.66, 95% CI 1.03–6.87).

In contrast, female sex was not significantly associated with mortality, despite a numerically lower hazard ratio, indicating that sex-related differences did not independently influence long-term survival after CTI ablation. This finding further supports the appropriateness of the CHA_2_DS_2_-VA score over sex-inclusive scores in this clinical context.

The independent prognostic value of non-CHA_2_DS_2_-VA factors in our model points to the possibility of constructing augmented risk scores. In other cardiovascular domains, CHA_2_DS_2_-VASc has been coupled with additional markers (e.g., renal function) to refine mortality prediction. For instance, Chen et al. found CHA_2_DS_2_-VASc to be an independent predictor of mortality in systolic heart failure populations, alongside renal dysfunction and thyroid disease (12). Future multicentre, prospective, large-scale registries should explore whether the integration of variables such as pulmonary disease, OSAS, and renal impairment improves discriminatory performance over CHA_2_DS_2_-VA alone. Moreover, this underscores the importance of holistic patient evaluation: attention to “nontraditional” risk factors might further refine prognosis and guide tailored interventions.

Taken together, our results suggest that CHA_2_DS_2_-VA scoring might aid post-procedural risk stratification and survival prediction of atrial flutter patients; refinement of prognostic models to incorporate key non-CHA_2_DS_2_-VA comorbidities could further improve predictive accuracy and guide long-term management strategies.

### Study limitations

This study has several limitations that should be acknowledged. First, it was a single-centre retrospective analysis, which inherently introduces the risk of selection bias and limits the generalizability of the findings to broader populations. Second, although the CHA_2_DS_2_-VA score was calculated at the time of ablation, changes in patients' clinical profiles during follow-up—such as newly developed comorbidities or progression of existing conditions—were not systematically assessed, which may have impacted long-term outcomes. Third, although multiple relevant comorbidities were included in the multivariate analysis, residual confounding from unmeasured variables cannot be completely excluded. Finally, small sample sizes in certain subgroups, particularly patients with OSAS, may have limited the precision of effect estimates and contributed to wide confidence intervals. Consequently, the observed association between OSAS and mortality should be interpreted cautiously and requires validation in larger cohorts.

Nevertheless, despite these limitations, the consistent association between the CHA_2_DS_2_-VA score and long-term mortality supports the robustness of our findings and underscores their potential clinical relevance in post-ablation risk stratification.

## Conclusions

In patients undergoing CTI ablation for typical AFL, the CHA_2_DS_2_-VA score emerged as a reliable and independent predictor of long-term all-cause mortality. A strong linear relationship between higher scores and increased mortality confirms its broader prognostic utility beyond thromboembolic risk assessment. Discriminatory performance demonstrates that the score meaningfully stratifies post-ablation survival risk throughout follow-up.

In addition, several comorbidities not encompassed by the CHA_2_DS_2_-VA score—particularly pulmonary disease and obstructive sleep apnoea—were identified as independent mortality predictors. These findings underscore the importance of comprehensive, individual evaluation of comorbid conditions when assessing long-term prognosis after flutter ablation. Future studies should aim to refine existing risk models by integrating such non-CHA_2_DS_2_-VA factors to enhance predictive accuracy and guide individualized follow-up strategies.

## Data Availability

The raw data supporting the conclusions of this article will be made available by the authors, without undue reservation.
