# Supplementary material for: CHA2DS2-VA and non-CHA2DS2-VA components as predictors of mortality after cavotricuspid isthmus ablation: a clinical tool for long-term risk stratification
Source: Front Cardiovasc Med. 2026 Jul 20;13:1884992. doi: 10.3389/fcvm.2026.1884992 (PMC13429838; doi:10.3389/fcvm.2026.1884992)
Supplement: Supplementary file 1 [file Table1.pdf]

Supplementary Table S1

Post-hoc pairwise log-rank comparisons between CHA_2DS_2-VA score categories using Holm-Bonferroni correction.

**Comparison**	**Adjusted p-value***
Score 1 vs Score 3	<0.001
Score 1 vs Score 4	<0.001
Score 1 vs Score 5	<0.001
Score 1 vs Score 6	<0.001
Score 1 vs Score 7	<0.001
Score 0 vs Score 4	0.021
Score 2 vs Score 4	0.038

*Only statistically significant pairwise comparisons after Holm–Bonferroni adjustment are shown.
